# Ecological Stoichiometry: A Link Between Developmental Speed and Physiological Stress in an Omnivorous Insect

**DOI:** 10.3389/fnbeh.2019.00042

**Published:** 2019-03-08

**Authors:** Giedrius Trakimas, Ronalds Krams, Tatjana Krama, Raine Kortet, Shahi Haque, Severi Luoto, Sarah Eichler Inwood, David M. Butler, Priit Jõers, Dror Hawlena, Markus J. Rantala, Didzis Elferts, Jorge Contreras-Garduño, Indrikis Krams

**Affiliations:** ^1^Institute of Biosciences, Vilnius University, Vilnius, Lithuania; ^2^Department of Biotechnology, Daugavpils University, Daugavpils, Latvia; ^3^Department of Plant Protection, Estonian University of Life Sciences, Tartu, Estonia; ^4^Department of Environmental and Biological Sciences, University of Eastern Finland, Joensuu, Finland; ^5^Institute of Ecology and Earth Sciences, University of Tartu, Tartu, Estonia; ^6^English, Drama and Writing Studies, University of Auckland, Auckland, New Zealand; ^7^School of Psychology, University of Auckland, Auckland, New Zealand; ^8^The Bredesen Center, Energy Science and Engineering, University of Tennessee, Knoxville, TN, United States; ^9^Department of Plant Sciences, University of Tennessee, Knoxville, TN, United States; ^10^Department of General and Microbial Biochemistry, University of Tartu, Tartu, Estonia; ^11^Department of Ecology, Evolution and Behavior, the Alexander Silberman Institute of Life Sciences, the Hebrew University of Jerusalem, Jerusalem, Israel; ^12^Department of Biology and Turku Brain and Mind Centre, University of Turku, Turku, Finland; ^13^Department of Botany and Ecology, Faculty of Biology, University of Latvia, Riga, Latvia; ^14^Ecuela Nacional de Estudios Superiores Unidad Morelia, Universidad Nacional Autónoma de México, Morelia, Mexico; ^15^Department of Psychology, University of Tennessee, Knoxville, TN, United States; ^16^Department of Zoology and Animal Ecology, Faculty of Biology, University of Latvia, Riga, Latvia

**Keywords:** carbon-to-nitrogen ratio, developmental speed, ecological stoichiometry, elemental body composition, trait-based ecology, *Gryllus integer*, pace-of-life syndrome, physiological stress

## Abstract

The elemental composition of organisms belongs to a suite of functional traits that may adaptively respond to fluctuating selection pressures. Life history theory predicts that predation risk and resource limitations impose selection pressures on organisms’ developmental time and are further associated with variability in energetic and behavioral traits. Individual differences in developmental speed, behaviors and physiology have been explained using the pace-of-life syndrome (POLS) hypothesis. However, how an organism’s developmental speed is linked with elemental body composition, metabolism and behavior is not well understood. We compared elemental body composition, latency to resume activity and resting metabolic rate (RMR) of western stutter-trilling crickets (*Gryllus integer*) in three selection lines that differ in developmental speed. We found that slowly developing crickets had significantly higher body carbon, lower body nitrogen and higher carbon-to-nitrogen ratio than rapidly developing crickets. Slowly developing crickets had significantly higher RMR than rapidly developing crickets. Male crickets had higher RMR than females. Slowly developing crickets resumed activity faster in an unfamiliar relative to a familiar environment. The rapidly developing crickets did the opposite. The results highlight the tight association between life history, physiology and behavior. This study indicates that traditional methods used in POLS research should be complemented by those used in ecological stoichiometry, resulting in a synthetic approach that potentially advances the whole field of behavioral and physiological ecology.

## Introduction

Ecological communities consist of a variety of species and are shaped by a complex array of intra- and interspecific interactions that maintain nutrient and energy flows through ecosystems (Meunier et al., 2017; Sperfeld et al., 2017). Importantly, for any given individual, the availability of resources in any particular environment is limited; time, effort and energy used for one purpose diminish those available for another (Stearns, 1992; Sperfeld et al., 2017). This often causes trade-offs in allocations of an individual’s resources to such competing life functions as immunity, reproduction, self-maintenance, development and growth (Roff, 1992; Krams et al., 2013a; Luoto, 2019).

Biotic and abiotic environmental stressors (e.g., predation, food limitation, extreme temperatures, drought, competition, growth in stressful conditions) may challenge organismal homeostasis (Boonstra, 2013; Wingfield, 2013; Ferguson et al., 2018). The stressed individual may then alter its behavior and functional traits to accommodate to the challenge, resulting in implications to their dietary choices and the elemental composition of their bodies and waste materials (Christianson and Creel, 2010; Hawlena and Schmitz, 2010a,b). This suggests that investments in stress tolerance and biochemical and behavioral adaptations to environmental stress may further affect the amount of energy available to each individual (Hochachka and Somero, 2002; Ellis and Del Giudice, 2014; Luoto, 2019). Ecological stoichiometry, a framework based on energetics, links the study of these trade-offs with the relative supply of elements in the environment and the metabolic demands and physiological traits of organisms (Meunier et al., 2017; Sperfeld et al., 2017).

Differences in behavioral and physiological responses to stress can be explained using the pace-of-life syndrome (POLS) hypothesis (Réale et al., 2010; Debecker and Stoks, 2018; Mathot and Frankenhuis, 2018; Royauté et al., 2018). This hypothesis originated from the classic concept of *r*- and *K*-selection (MacArthur and Wilson, 1967; Pianka, 1970) and the more recent idea of fast-slow life history continuum (e.g., Promislow and Harvey, 1990; Bielby et al., 2007). It suggests that differences in life history strategies among species or populations are associated with physiological (e.g., metabolic rate; Ricklefs and Wikelski, 2002; Wikelski et al., 2003; Wiersma et al., 2007) and behavioral differences (Wolf et al., 2007; Biro and Stamps, 2008; Réale et al., 2009; Luoto, 2019). The POLS hypothesis predicts that rapidly developing individuals with high activity and boldness have faster life histories (e.g., faster development and reproduction) and higher metabolic rate, which reduces life span through increased oxidative damage (Janssens and Stoks, 2018). This prediction is based on the assumption that high activity and boldness increase resource acquisition. Passive and shy individuals, in contrast, are expected to show the opposite features (Réale et al., 2010).

However, evidence suggests that slower development of prey individuals may also incur stress as it is associated with upregulation of stress-related genes (Gutiérrez-Adán et al., 2004). Wings of late-hatched female damselflies *Lestes viridis* were found to be more asymmetrical than those of early-hatched ones (De Block et al., 2008). Importantly, selection for slower development confers higher levels of anxiety/neuroticism along the stress reactivity axis in crickets (Krams et al., 2017). The observed anxiety in behavior and resting metabolic rate (RMR) in slowly developing crickets under stressful conditions decreased after a selective serotonin reuptake inhibitor (SSRI) treatment (Krams et al., 2018). On the other hand, slower development may be associated with longer lifespan (Brooks and Garratt, 2017; Kecko et al., 2017) which may require an improved immune system (Niemelä et al., 2013; Krams et al., 2015, 2016a). In some species where females have longer lifespan than males, the strength of immune responses and inflammatory immune responses are generally higher in females than in males (Klein, 2012; Klein and Flanagan, 2016; Kecko et al., 2017). However, females often suffer a higher propensity to many autoimmune diseases such as rheumatoid arthritis, fibromyalgia, anxiety and depression (e.g., Dumont-Lagacé et al., 2015), suggesting associations between slower development, longer lifespan and stress resistance (Brooks and Garratt, 2017).

It is considered that rapidly developing individuals are bolder and more stress resistant than slowly developing shy individuals (e.g., Steimer et al., 1997). Nevertheless, how an organism’s developmental speed is linked with elemental body composition, metabolism and behavior is not well understood, and a comprehensive approach that combines stoichiometry with behaviors has been lacking in prior research. Selective lines is an effective method to produce comparable individuals of varying developmental times (Krams et al., 2017). Here, we tested behavioral responses to handling (as a proxy of stress resistance; Adamo et al., 2013), RMR and elemental body composition of three selected lines of western stutter-trilling crickets (*Gryllus integer*) that differ in developmental speed.

Based on existing findings on the effects of stress on organismal stoichiometry (Hawlena and Schmitz, 2010a,b; Krams et al., 2016b), we predicted higher concentrations of carbon (C), lower nitrogen (N), a greater C/N ratio and greater RMR as indicators of physiological stress in the slow developmental line compared with the rapid line and possibly also with the control line because of the higher sensitivity to antidepressants (selective serotonin reuptake inhibitor, SSRI) found in the slow and control developmental lines (Krams et al., 2018). We predicted shorter latency of resuming activity among startled slowly developing individuals (as opposed to rapidly developing crickets) in an unfamiliar environment as an indicator of physiological stress. It has been shown that SSRI treatment increased the time to resume movements (i.e., decreased anxiety) of slowly developing crickets in an unfamiliar environment (Krams et al., 2018). Since males and females may differ in their stress responses, we tested for possible sexual dimorphism in concentrations of C, N and the C/N ratio (Bayer and Hobert, 2018).

## Materials and Methods

### Insects and Selection Lines

The laboratory stock originated from a wild population (Davis, CA, USA). This stock was first maintained at the University of Oulu and the University of Eastern Finland, and then moved to the University of Tartu in Estonia, where the present data were collected. In this study, we tested crickets that had been selected for five generations for their developmental speed. *G. integer* nymphs were reared individually in plastic containers (28 × 98 × 73 mm: length, width, height, respectively) with a hole of 30 mm in diameter covered with plastic netting for ventilation. Each container was equipped with a shelter made of cardboard. The individual crickets were kept under a constant 12:12 h light–dark cycle, at 26 ± 1°C with *ad libitum* food consisting of fish flakes (Eheim) and reindeer pellets (Rehuraisio Oy, Poron herkku) and *ad libitum* water. Although nymphal density does not affect adult behavior, it does increase life history investments in immune function and maturation (Niemelä et al., 2012b).

The selection design consisted of three main selection lines (rapid development, slow development and control). In each generation, offspring were obtained from ~20 families within each main line. For rapid and slow developmental lines, mated males and females were selected according to their maturation time, and only the most rapid or slowest maturing individuals were used for matings in each main line (for more details on selection, see Krams et al., 2017). In the control line, matings were randomized over the whole natural maturation time range. Two months after hatching, random samples of offspring from the rapid developmental line were placed into individual containers in a random order. The same procedure was performed 3 months after hatching in the control line and 4 months after hatching in the slow line in each generation.

After five generations of selection for developmental speed, developmental time (the average maturation time ± SD) for rapidly developing individuals was 91.03 ± 6.06 days (*n* = 29 crickets), 117.33 ± 7.53 days (*n* = 30) for the control individuals and 136.17 ± 8.28 days (*n* = 24) for slowly developing crickets. All groups differed significantly in their developmental time [one-way analyses of variance (ANOVA): *F*_(2,77)_ = 257.89, *P* < 0.0001].

### Body C and N Content

Following food deprivation of 15 h and water *ad libitum*, all crickets were immediately frozen at −80°C (Angelantoni Lifescience, Italy). Before elemental analysis, we dried bodies of 29 rapidly developing crickets, 30 control crickets and 24 slowly developing crickets at 60°C for 48 h. Each individual was ground to a homogenous powder and measured for C and N content using a C-N combustion auto-analyzer (Hawlena and Schmitz, 2010a,b; Krams et al., 2016b).

### Behavioral Trials: Resuming Activity in a Familiar Environment

We started behavioral trials on day 10 after crickets reached maturity. Before behavioral trials and measuring RMR, we weighed each individual using a Kern analytical balance (ABT 120-4M; Kern and Sohn, Balingen, Germany). Behavioral trials were conducted under constant temperature (25 ± 1°C) and sound-proof conditions. We used dim red light (25 W red incandescent bulb) since *Gryllus* spp. cannot see long (red) wavelengths properly (Briscoe and Chittka, 2001), which allowed us to observe these nocturnal insects without disturbing them. The crickets were provided with drinking water before the onset of the trials, while food was removed 5 h before the beginning of experimental trials.

We captured the focal cricket in its housing-box and handled it by holding the insect in the hand for 1 min. After the handling procedure, the cricket was placed back in its burrow-like triangular cardboard shelter (5 cm long, with a 1 × 1 × 1 cm entrance). We recorded the latency to resume activity when the insect started to move inside the cardboard shelter. We waited for all crickets to resume activity (max. 908 s). The same procedure was repeated for each individual 4 days later. Latency to resume activity indicates the duration of freezing or immobile state, a widespread anti-predator response occurring in many taxa (Chelini et al., 2009; Krams et al., 2013a,b).

### Behavioral Trials: Resuming Activity in an Unfamiliar Environment

Two days after the first test conducted in an environment familiar to the crickets, we handled them for 1 min and then gave them the opportunity to escape into a burrow-like conical plastic Eppendorf test tube (volume 5 mL, Sigma-Aldrich), which was used as an insect chamber. The insect chamber was connected to the respirometer with rubber tubing (Lighton, 2008). When reaching the insect chamber, each of the crickets immediately became completely immobile, as if it was hiding in a burrow. We waited for all crickets to resume active struggling movements (max. 1850 s).

### Metabolic Rate Measurements

We measured cricket RMR as the rate of carbon dioxide emission in an incurrent flow-through system. The LI-7000 differential CO_2_/H_2_O analyzer (LiCor, Lincoln, NE, USA) was calibrated at different flow rates by means of calibration gases (Trägergase, VEB, Saxon Junkalor GmbH, Dessau; Quinlan and Lighton, 1999; Lighton, 2008) with gas injection (see also Kuusik et al., 2002; Mänd et al., 2006). While measuring CO_2_ emissions, the insect chamber was perfused with dry (5**–**7%RH) CO_2_-free air, produced by passing air over Drierite (W. A. Hammond Drierite Co. Ltd., Xenia, OH, USA) and soda-lime granules at an airflow rate of 60 ml min^−1^. Average ambient temperature within the respirometry chamber was 23.60 ± 0.30°C. Baseline drift of the analyzer was corrected during analysis from the measurements at the beginning and end of each trial with the respirometer chamber empty (Duncan, 2003; Duncan and Byrne, 2005; Gray and Bradley, 2006). The respirometric device was combined with an infrared optical system using IR emitting diodes (TSA6203) and IR-sensor diodes (BP104) that were placed on the sides of the insect chamber. IR-diodes made it possible to record CO_2_ production and to follow movements of each cricket simultaneously. The insects remained in their chambers for 4 h, and we recorded their minimum rates of metabolism at moments when crickets were immobile. As soon as the measurements were over, we returned the crickets back to their plastic housing-boxes. We repeated the trials 5 days later. Since insects differed in their body mass, we used body mass-specific RMR values in this study.

### Statistical Analyses

We used two-way ANOVAs with developmental line (slow, rapid, control) and sex as fixed factors to assess differences in elemental composition (C, N and C/N ratio). We considered elemental composition analyses to belong to the same families of tests (Rubin, 2017). We thus controlled for multiple testing using Holm-Bonferroni procedure to adjust *P*-values. An adjusted *P*-value < 0.05 was considered to be statistically significant. Assumptions of homogeneity of variances were met (Levene’s test, *P* > 0.05). We report only the main effects when no significant interactions between fixed factors were found; otherwise, Tukey’s HSDs are also reported. To test for the effects of developmental line and sex on RMR, linear mixed effects model (LMM) was used. Another LMM was fitted to test for the effects of developmental line, sex and environment (familiar, unfamiliar) on differences in latency to resume activity (log-transformed). In both LMMs, individual cricket ID was included as a random factor to account for the possible correlation of repeated measurements of the same individual. Analyses were performed using IBM SPSS 22 for Windows and the program R, version 3.3.2 (R Development Core Team, 2016).

## Results

### Carbon

**Figure 1:**
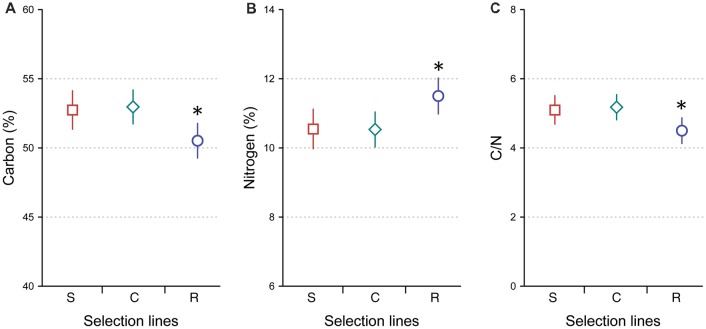
Average carbon percentage **(A)**, nitrogen percentage **(B)** and carbon-to-nitrogen ratio **(C)** in *Gryllus integer* crickets selected for slow development (S, squares, *n* = 24), rapid development (R, circles, *n* = 29) and control developmental (C, diamonds, *n* = 30) lines. Error bars represent 95% confidence intervals. Asterisks indicate significant differences between the lines (*P < 0.05).

Rapidly developing crickets had less body C (%; 50.5 ± 3.5, mean ± SD) than slowly developing (52.7 ± 3.0, mean ± SD) and control (53.0 ± 3.5, mean ± SD) crickets (Tukey’s tests, *P* = 0.047 and *P* = 0.02, respectively), while slowly developing and control crickets did not differ in body C (Tukey’s test, *P* = 0.96; Figure 1A). The main effect of developmental line to body C was significant (two-way ANOVA: *F*_(2,77)_ = 4.562, *P* = 0.039). Body C of females and males did not show significant differences (two-way ANOVA: *F*_(1,77)_ = 0.962, *P* = 0.33). There was no significant interaction between developmental line and sex to body C (two-way ANOVA: *F*_(2,77)_ = 0.484, *P* = 0.618).

### Nitrogen

In contrast, rapidly developing crickets had higher body N (%; 11.5 ± 1.4, mean ± SD) than slowly developing (10.6 ± 1.2, mean ± SD) and control (10.5 ± 1.5, mean ± SD) crickets (Tukey’s tests, *P* = 0.031 and *P* = 0.022, respectively). Slowly developing and control crickets did not differ in body N (Tukey’s test, *P* = 1.0; Figure 1B). The main effect of developmental line to body N was significant (two-way ANOVA: *F*_(2,77)_ = 4.452, *P* = 0.03), while sex had no effect on it (two-way ANOVA: *F*_(1,77)_ = 1.062, *P* = 0.306). There was no significant interaction between developmental line and sex to body N (two-way ANOVA: *F*_(2,77)_ = 0.368, *P* = 0.693).

### C/N Ratio

Rapidly developing crickets had a lower C/N ratio (4.48 ± 0.91, mean ± SD) than control crickets (5.18 ± 1.10, mean ± SD; Tukey’s test, *P* = 0.028). A marginally non-significant difference was found when comparing rapidly developing crickets with slowly developing crickets (5.1 ± 0.94, mean ± SD; Tukey’s test, *P* = 0.068). Slowly developing and control crickets did not differ in their C/N ratio (Tukey’s test, *P* = 0.98; Figure 1C). The main effect of developmental line was significant (two-way ANOVA: developmental line: *F*_(2,77)_ = 3.904, *P* = 0.024), while C/N ratios of females and males were not significantly different (two-way ANOVA: *F*_(1,77)_ = 1.144, *P* = 0.288). There was no significant interaction between developmental line and sex to C/N (two-way ANOVA: *F*_(2,77)_ = 0.229, *P* = 0.796).

### Resting Metabolic Rate

**Figure 2:**
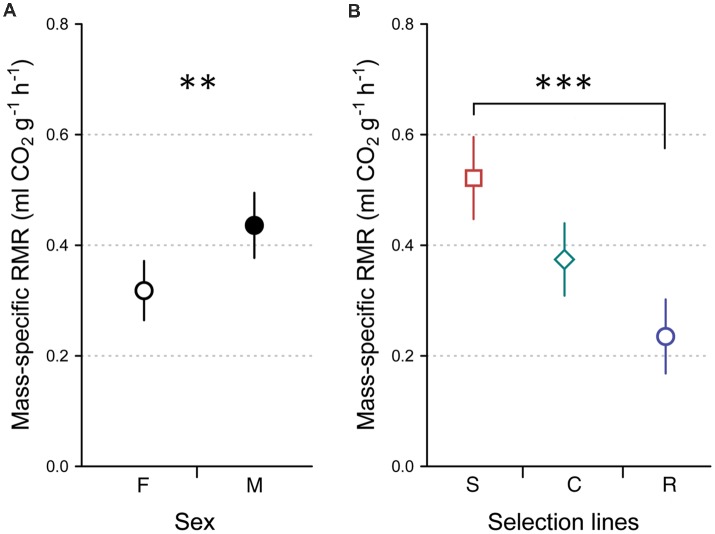
Mass-specific resting metabolic rate (RMR) of females (F, open circle, *n* = 45) and males (M, closed circle, *n* = 38; **A**), selected for slow development (squares, *n* = 24), rapid development (circles, *n* = 29) and control developmental (diamonds, *n* = 30; **B**) lines. Symbols and error bars show averages and 95% CIs, respectively. Asterisks indicate significant differences (**P < 0.01; ***P < 0.001).

Body mass-specific RMR differed significantly between females and males (LMM, *F*_(1,77)_ = 8.704, *P* = 0.004), with male crickets having higher RMR than females (Figure 2A), and between developmental lines (LMM, *F*_(2,77)_ = 16.329, *P* < 0.0001). The highest mean RMR was recorded in crickets from the slow developmental line and the lowest RMR was recorded in crickets from the rapid developmental line (Figure 2B). We found no significant interaction between developmental line and sex (LMM, *F*_(2,77)_ = 0.430, *P* = 0.652).

### Resuming Activity in Familiar and Unfamiliar Environments

**Figure 3:**
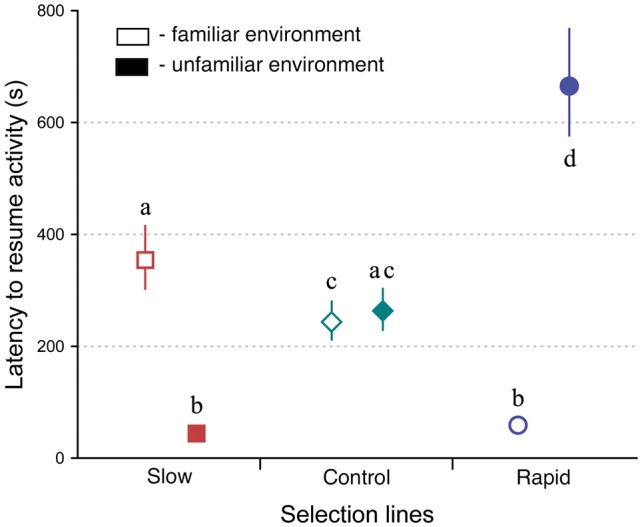
Latency to resume activity (back-transformed means ± 95% CIs) of selected slow (squares), rapid (circles), and control (diamonds) developing crickets in familiar (open symbols) and unfamiliar environments (closed symbols). Different letters denote significant differences at *P* < 0.05.

Selection line and environment had significant effects on crickets’ behaviors (LMM, *F*_(2,332)_ = 44.663, *P* < 0.0001, and *F*_(1,332)_ = 5.319, *P* = 0.022, respectively), while sex had no effect (LMM, *F*_(1,332)_ = 0.778, *P* = 0.378; Figure 3). There was also a significant interaction between selection line and environment (LMM, *F*_(2,332)_ = 452.9, *P* < 0.0001). Thus, slowly developing crickets resumed activity faster in an unfamiliar but more slowly in a familiar environment on average, while rapidly developing crickets did the opposite. Other interactions were respectively non-significant and marginally non-significant: sex*environment (LMM, *F*_(1,332)_ = 0.486, *P* < 0.486) line*sex (LMM, *F*_(2,332)_ = 3.01, *P* = 0.051).

## Discussion

Our results show that differences in POLS (developmental speed) alter insect body stoichiometry and that those changes were associated with different behavioral and physiological stress responses. Thus, developmental speed should be considered as an important factor in research on ecological stoichiometry as it may influence the need for specific nutrients (see Snell-Rood et al., 2015; Camus et al., 2017) and POLS trait interactions more generally.

Surprisingly, slowly developing crickets did not differ significantly from the control line on any of the three stoichiometric parameters while rapidly developing crickets did on all three of them. Rapidly developing crickets had the lowest C, the highest N concentrations and the lowest C/N ratio. This suggests that rapidly developing crickets probably experienced the lowest levels of stress during their nymphal development or were more resilient to stress because of genetic or epigenetic factors. Rapidly developing individuals should benefit from faster growth because this provides a higher chance of surviving to reproduction at low cost (Roff, 1992; Stearns, 1992). This finding is contradictory with predictions arising from life history theory because it has been traditionally assumed that juvenile growth rates operate near their physiological maximum (Stearns and Koella, 1986; Roff, 1992; Stearns, 1992) where tight energy budgets may bring oxidative stress and other costs (Fischer et al., 2004; Slos and Stoks, 2008; Krams et al., 2017). A number of studies confirm that rapid growth is costly, which suggests that growth rates are generally optimized rather than maximized in many species (Gotthard et al., 1994; Nylin et al., 1996; Lankford et al., 2001; Arendt, 2003; Fischer et al., 2004). However, we show that slowly developing and control crickets may be under higher levels of stress as indicated by their higher body C concentration, lower body N and higher C/N ratio. This may be especially true in an unfamiliar environment where slowly developing and control crickets were found to have higher RMR than rapidly developing crickets.

These results can be framed in a larger context of ecological stoichiometry by considering an interesting set of results reported in another species. Grasshoppers (*Melanoplus sanguinipes*) from a subarctic region have about 80 days shorter growing (rapid growth) season compared to those from temperate areas (Fielding and Defoliart, 2007). This shorter growth period is associated with decreased body mass of the subarctic grasshoppers but improved post-ingestive efficiencies and N assimilations on low- and high-quality foods. The temperate grasshoppers, in contrast, are less efficient in digesting low-quality food. This shows that faster development may not generally affect digestive abilities of orthopterans and are unlikely to be associated with elevated levels of physiological stress.

The current results on behavioral traits support an earlier study which showed that slowly developing crickets (compared with rapidly developing ones) resume their daily activities more slowly after being handled in a familiar (less stressful) environment and faster when being handled in a novel (more stressful) environment (Krams et al., 2017). This suggests that slowly developing, often shy crickets are more stressed than rapidly developing, often bold ones. Previous studies show that slowly and rapidly developing crickets markedly differ in behavioral and physiological traits: slowly developing individuals are shy, generally larger, more stressed under unfamiliar conditions, they show stronger encapsulation responses and have higher RMR and lower maximum metabolic rate (MMR) compared with rapidly developing crickets (Niemelä et al., 2012a, 2013; Krams et al., 2017).

Although field cricket females are bigger in body size than males and can occasionally kill them (Kortet and Hedrick, 2005), we did not find any signs of higher stress in males. It is likely that the higher dominance position of female crickets is outweighed by their higher somatic and reproductive costs because a significant correlation between female body size and fecundity is often observed in *G. integer* and other insects (Blanckenhorn, 2000; Hedrick and Kortet, 2012; but see Tammaru et al., 2002). It is important to note that crickets lived in individual cages in this study so that males and females could not interact with each other. We hypothesize that living in groups would increase stress in males, which needs to be tested by measuring behavioral reactions, concentrations of hormones and metabolic rate coupled with research methods used in ecological stoichiometry.

While it is possible that hormone concentrations in the brains of slowly developing crickets serves as a proximate mechanism underlying their higher stress levels and differences in body elemental composition (Stevenson et al., 2005; Zhou et al., 2008; Adamo and McKee, 2017; Krams et al., 2018), it is not clear what is the ultimate reason for becoming shy when developing slowly. Intuitively, shy individuals may benefit from limited activities and greater suspiciousness under higher predator risk. However, it is not clear why this adaptation should be reached *via* a stressed phenotype (e.g., higher C, higher RMR, lower N, slower development). Acquiring a more holistic understanding of these questions would be facilitated by studying crickets’ physiological condition, body elemental composition, neurotransmitter concentrations, antipredator responses, sexual selection and survival under natural conditions to test whether shy personality always co-occurs with elevated anxiety and heightened physiological markers of stress. Future studies should include an assessment of phosphorus concentration which is important for RNA production and serves (quantified as the RNA:DNA ratio) as a proxy for protein synthesis (Janssens et al., 2017). These approaches are needed to further develop the general stress paradigm (Hawlena and Schmitz, 2010a).

## Conclusion

The results of this study show that slow development is associated with a stressed phenotype. This phenotype is characterized by shorter behavioral latencies in a novel environment and higher stress levels associated with higher body C and lower N concentrations. This study shows that ecological stoichiometry is a tool that needs to be used alongside other traditional methods to study animal stress. Explicit focus on ecological stoichiometry has the potential to explain contradictory results, to sharpen predictions and to move the general stress research paradigm forward through a more holistic understanding of organismal responses to fluctuating selection pressures.

## Author Contributions

GT, IK and RKo: conceptualization. IK, TK, RKr, SH, RKo and MR: designed the methodology. DE, GT, JC-G and IK: performed the formal analysis. SEI, DB, TK, RKr, IK, PJ and MR: performed the experiments. IK, GT, SH and SL: wrote the original draft. TK, RKo, RKr, SEI, DB, DH, PJ, MR, JC-G and SL: wrote, reviewed and edited the submitted version. GT designed the figures. IK and TK: responsible for funding acquisitions, supervision and administration of the project.

## Conflict of Interest Statement

The authors declare that the research was conducted in the absence of any commercial or financial relationships that could be construed as a potential conflict of interest.
